# Bypassing health facilities for childbirth in the context of the JSY cash transfer program to promote institutional birth: A cross-sectional study from Madhya Pradesh, India

**DOI:** 10.1371/journal.pone.0189364

**Published:** 2018-01-31

**Authors:** Yogesh Sabde, Sarika Chaturvedi, Bharat Randive, Kristi Sidney, Mariano Salazar, Ayesha De Costa, Vishal Diwan

**Affiliations:** 1 Department of Community Medicine, R.D. Gardi Medical College, Ujjain, India; 2 Department of Public Health Sciences, Karolinska Institutet, Stockholm, Sweden; 3 Department of Public Health and Environment, R.D. Gardi Medical College, Ujjain, India; 4 Epidemiology and Global Health, Department of Public Health and Clinical Medicine, Umea University, Umea, Sweden; 5 International Centre for Health Research, R.D. Gardi Medical College, Ujjain, India; Public Library of Science, UNITED KINGDOM

## Abstract

Bypassing health facilities for childbirth can be costly both for women and health systems. There have been some reports on this from Sub-Saharan African and from Nepal but none from India. India has implemented the Janani Suraksha Yojana (JSY), a large national conditional cash transfer program which has successfully increased the number of institutional births in India. This paper aims to study the extent of bypassing the nearest health facility offering intrapartum care in three districts of Madhya Pradesh, India, and to identify individual and facility determinants of bypassing in the context of the JSY program. Our results provide information to support the optimal utilization of facilities at different levels of the healthcare system for childbirth. Data was collected from 96 facilities (74 public) and 720 rural mothers who delivered at these facilities were interviewed. Multilevel logistic regression was used to analyze the data. Facility obstetric care functionality was assessed by the number of emergency obstetric care (EmOC) signal functions performed in the last three months. Thirty eighth percent of the mothers bypassed the nearest public facility for their current delivery. Primiparity, higher education, arriving by hired transport and a longer distance from home to the nearest facility increased the odds of bypassing a public facility for childbirth. The variance partition coefficient showed that 37% of the variation in bypassing the nearest public facility can be attributed to difference between facilities. The number of basic emergency obstetric care signal functions (AOR = 0.59, 95% CI 0.37–0.93), and the availability of free transportation at the nearest facility (AOR = 0.11, 95% CI 0.03–0.31) were protective factors against bypassing. The variation between facilities (MOR = 3.85) was more important than an individual’s characteristics to explain bypassing in MP. This multilevel study indicates that in this setting, a focus on increasing the level of emergency obstetric care functionality in public obstetric care facilities will allow more optimal utilization of facilities for childbirth under the JSY program thereby leading to better outcomes for mothers.

## Background

Bypassing health facilities for care is a phenomenon where individuals choose to obtain care from a facility that is not their nearest [1]. Studies conducted in low- and middle-income countries found that the magnitude of bypassing facilities for any type of care ranged from 36% in Chad to 67% in Tanzania [1,2]. In the USA, analyses of rural populations´ bypassing patterns found that between 37% and 44% of patients bypassed their local hospital for care [3,4].

Research from low- and middle-income settings have highlighted that pregnant women and their families often circumvent their nearest obstetric care facility for childbirth. In Tanzania and Nepal, 44% and 70% of the pregnant women respectively bypassed their nearest facility for childbirth [5,6]. The magnitude of bypassing can vary significantly even within settings. In Uganda, Parkhurst and Sengooba found that between 8–80% of the facility births attributable to a parish were attended by the parish local obstetric facility [7].

Though many health systems in low- and middle-income settings are tiered, not all have formal gate keeping functions. Tiered obstetric health services are important because non-complicated cases can be handled in primary care settings and complicated cases can be centralized in secondary and tertiary settings [8].

From the health system perspective, bypassing has adverse effects both for lower and higher level facilities. Bypassing lower level facilities for childbirth can disrupt quality of care as higher level facilities become overcrowded [8]. Overcrowding stretches secondary and tertiary hospitals´ human and logistic resources to the limit, impairing the efficiency of these facilities to effectively provide care of an adequate standard and thereby reduce maternal morbidity/mortality [9]. Non-utilization of lower tiered facilities can lead to loss of existing levels of emergency obstetric care functionality.

From the users´ perspective bypassing can have negative and positive consequences. It might increase travel expenses, and time to reach a health facility. However, it might also mean accessing facilities with better functionality which might translate into better care.

Maternal mortality is a significant public health problem in India. As per the global burden of disease report 2013, the country contributed 24% (71792/ 292982) of all global maternal deaths [10]. In order to increase pregnant women´s access to facility based childbirth, and thereby reduce maternal mortality, the government of India implemented a nationwide, conditional cash transfer program, Janani Suraksha Yojana (JSY) [11]. Under the program, women who give birth in public facilities receive a cash transfer on discharge from the facility. Since its implementation in 2005, the program has had 63 million beneficiaries [12]. Hospital delivery rates in India have risen steeply from 38% in 2005 to 74% in 2013 [13]. Notwithstanding its success, the program has not been associated with reduced maternal mortality in this setting [14].

In spite of the substantial resources invested by the Indian government to implement the program, and the significant increase in institutional deliveries after the introduction of the JSY [15] there are no studies assessing the extent of bypassing public obstetric care facilities for childbirth. The extent of bypassing has implications for the JSY program, it provides feedback of program uptake at different levels of facilities, and therefore is indicative of deficiencies of functioning at particular levels—including underutilization at lower levels (and thereby risking a drop in the ability to provide emergency obstetric care functions at those levels) and an overutilization at higher level facilities (risking a drop in the quality of care provided at these levels).

In this paper, we therefore aim to study the phenomenon of bypassing for childbirth in the context of the JSY program in the Indian state of Madhya Pradesh (MP). Specifically, this paper aims to 1) study the extent of bypassing of public obstetric care facilities among rural mothers in three districts of MP, India 2) to identify characteristics of obstetric care (OC) facility that result in it being bypassed or utilized by rural mothers 3), and to identify the characteristics of women who bypassed the nearest OC facilities. Also, we assess the relative contribution of maternal and facility characteristics to bypassing.

## Methods

### Study setting

The study was conducted in MP state, India. MP has a population of 72 million (48% women), 72% living in rural areas [16]. Thirty percent of the population is illiterate (45% among women) [16]. MP is divided into 51 administrative units called districts, each with population about one to two millions. The infant mortality rate (IMR) and maternal mortality ratio (MMR) (65 per 1,000 live births and 277 per 100,000 live births, respectively) in MP are amongst the highest in India [17].

Three different districts were purposively selected so that they represented distinct geographic areas of the state, different cultural zones, and had different population compositions—one was a relatively urban district, the second a tribal forested district and the third a rural district. The three districts also represented different levels of socioeconomic development, as measured by their IMR, MMR and human development index (details are shown in Table 1).

**Table 1:** Profile of the study districts.

Characteristics	District 1	District 2	District 3
Population (millions)*	1.99	1.02	1.07
Maternal mortality ratio*	268	435	397
Human development index†	0.6	0.5	0.5
Proportion (%) of institutional deliveries ‡	81	72	59

* Government of India (2011) Provisional Population Totals: Madhya Pradesh Census.

^†^Government of Madhya Pradesh, Madhya Pradesh Human Development Report 2007, Government of Madhya Pradesh: Bhopal.

^‡^Registar General of India, Annual Health Survey of India 2012, Government of India: New Delhi.

In MP, the public sector is the dominant provider of facility based intrapartum care. The private sector operating on fees for service paid out of pocket is small and concentrated in urban areas. The public health care delivery system as in the rest of India has three tiers. The JSY program is implemented at all tiers of the system.

In this system, sub-centers (SCs) and primary health centers (PHC) are primary points of care. Located at village level they intend to serve a population of 5,000 and 30,000 respectively, though catchment populations are often higher in reality. The PHCs/ SCs are expected to provide normal delivery care and referrals when there are complications. A few (but not the majority of) PHCs provide obstetric care 24/7. Sub-centers, which are community health outposts under a PHC, have extremely varying levels of functionality. Many are open only on particular days and most do not have a fully functional labor room.

Secondary level facilities are known as community health centers and sub-district hospitals (CHC/SDH). Located at sub-district level they cater to an approximate population of 100,000. CHCs are expected to provide specialist services (though many do not). A few of these are designated as First Referral Units (FRUs) providing Caesarean sections (CS). District hospitals (DH) are expected to be tertiary care facilities handling complicated cases and able to provide both CS and blood transfusion. Women are free to choose any facility they wish to give birth in; there are no strict gatekeeping functions observed.

The median volume of births in the public sector varies depending on their level of emergency obstetric care (EmOC) functionality. In a previous study in this setting, we found in that facilities that provide less than the seven basic EmOC signal functions, the median number of births over a three months period was 41 (IQR 11–115), while in those providing CS services the median number of births in the same period was 658 (IQR 642–1188)[18].

The JSY program in MP is open to all women, regardless of parity, poverty status, tribal status or antenatal care received. The state has seen the highest JSY uptake in the country. In 2009, 86% of women in MP were aware of the JSY program, and 72% had institutional deliveries [19]. Women delivering at any level of public health facility are eligible to receive the cash transfer. Under the JSY program [20], pregnant women have access to support provided by an accredited social health activist (ASHA), a locally community-based village female volunteer, who receives an incentive from the state to facilitate institutional births under the program [21]. In addition, they have access to the Janani Express (JE), an emergency free transport service to transport pregnant women from their homes to a public health facility [22].

### Study type, sample size and sampling procedures

A cross-sectional health facility survey of all facilities in the study area was conducted between February 2012 and April 2013. A list of all health facilities (public and private) that provided intrapartum care in each district was obtained from the district level health authorities. For the purpose if this study, obstetric care (OC) facilities were defined as those that had done at least 30 deliveries in the last three months. A total of 99 facilities (74 public) were eligible to participate and out of those, three private facilities declined.

All women giving birth in these facilities over a five consecutive day period were approached for participation (n = 1122). Of those, 14 women refused to participate, 175 were excluded from the study (79 were referred from others facilities, 89 resided outside the study districts), and seven had missing address details).

Since this study focuses on assessing the factors associated with rural mothers bypassing their nearest public health care facility for childbirth in the context of the JSY intervention, women whose nearest facility was private (n = 80) or those who lived in an urban setting (n = 133) were excluded from analysis. The final sample was 720 women.

### Data collection

After identification, all facilities were visited by trained women research assistants who collected information from two levels, (i) facility level characteristics from the facility administration, and (ii) all women delivering in these facilities over the five consecutive day period.

Facility characteristics studied included obstetric care functionality. This was assessed by eliciting information about the basic (BEmOC) and comprehensive emergency obstetric (CEmOC) care signal functions performed in the last three months [23]. Trained researchers met with physicians/ nurses leading the facility or the obstetric services (in larger hospitals) to obtain this information. These data was cross-checked with the facility’s records.

Obstetric signal functions were defined as in the UNFPA handbook [23] “*key medical interventions that are used to treat the direct obstetric complications that cause the vast majority of maternal deaths around the world*”[23]. BEmOC functions included: 1.administration of parenteral antibiotics, 2. administration uterotonic drugs, 3. administration parenteral anticonvulsants for preeclampsia/eclampsia, 4.manual remove of placenta, 5.removal of retained products of contraception, 6.assisted vaginal delivery, and 7.neonatal resuscitation. A facility was classified as BEmOC competent if it has performed all seven basic functions in the last three months. A CEmOC competent facility will have performed all the seven basic functions in addition to the two special functions of C-section and blood transfusion [23]. Other facility data collected included general characteristics such as ownership and availability of free transportation.

Maternal data elicited included socio-demographic (age, education, caste and ownership of below poverty line card) and detailed obstetric history linked to the current childbirth. The former included: parity, number of antenatal care (ANC) visits during current pregnancy, type of transportation used to reach OC facility, complications during childbirth (bleeding, high blood pressure, obstructed/prolonged labor, convulsions, ruptured uterus, infection, and retained placenta), address and if the ASHA accompanied the woman to the OC facility for childbirth.

Maternal rural residency was defined following the terms used by Indian Censuses: “*Rural areas comprise of revenue villages which are the smallest areas of habitation*. *The revenue village generally follows the definite surveyed boundary that is recognized by the district administration*. *It may have one or more hamlets but the entire revenue village is one unit*. *There may be unsurveyed villages within forests etc*., *where the locally recognized boundaries of each habitation area is followed within the larger unit of say the forest range officer’s jurisdiction*” [24].

### Mapping to identify the closest facility and defining bypassers

The outcome variable, bypassing, was defined as pregnant women who did not deliver at the nearest OC facility from their village of residence [1]. The construction of this variable was as follows: 1. Survey of India topographical maps of the scale 1:50,000 were used for geo-referencing OC facilities and mother’s villages (identified from their address); 2. Using the geo-referenced data, network analysis tool in ArcMap 10 was used to identify the nearest OC facility for the each mothers’ village [25]. The distance to the actual facility where she gave birth was then calculated. If this latter distance was greater than the distance to the nearest facility (from her home); she was classified as a bypasser.

### Analysis

A database was created using research electronic data capture (REDCap) [26]. Primary data was entered into REDCap and subsequently exported to STATA version 12 for analysis. Percentages, means, medians, and interquartile range (IQR) were used to describe maternal characteristics. Chi square, t-test, and Kruskal-Wallis test were used to test for significant differences between groups when appropriate

A multilevel mixed effects logistic regression model was chosen to identify the relative importance that contextual factors (OC facility factors) have in understanding the variation in bypassing in this setting [27]. The multilevel mixed effects logistic regression allowed us to account for the clustered nature of our data.

A continuous variable describing the number of basic signal functions was created to facilitate analysis. C-section and blood transfusion were considered key signal functions of CEmOC [23], thus were introduced separately in the analysis. The continuous BEmOC variable was highly correlated with the facility type (level) variable, thus the latter was excluded from the model.

Individual and facility variables that had a significant association with the outcome (p<0.05) in the bivariate analysis were included in the multilevel models. The multilevel analysis is presented in three consecutive models. First, an empty model that only included a random intercept to assess if the occurrence of the bypassing phenomenon was influenced by differences between the nearest OC facilities. The second model added individual-level variables to determine if OC facilities differences were due to variations in women’s characteristics within each nearest OC facility. The third model included both individual-level and facility-level variables to assess if bypassing was associated with specific facility level characteristics. Multicolinearity was defined as variable inflation factor values equal to or greater than 10, or tolerance values below 0.1. No multicolineality between the variables was found.

Two measures of variation between OC facilities were used. The variance partition coefficient (VPC) represented the proportion of the variance (VA) that was due to the difference between facilities [28]. The median odds ratio (MOR) is defined as the median value of the odds ratio between the facility at highest risk (of being bypassed) and the facility at lowest risk when randomly picking out two facilities [28]. The median odds ratio can be used to quantify facility level effects on individual health behavior (bypassing) [28]. In our study, the MOR shows the extent to which a woman´s probability of bypassing her nearest facility is determined by the characteristics of that facility. Variables with p value < 0.05 in the final multivariate model were considered significant.

We conducted a sample size calculation for our cross-sectional study using the following parameters: 95% confidence interval, 40% bypassing prevalence, population size 1,000,000, and design effect 1.8. This analysis gave us a sample size of 664; thus our sample size of 720 was deemed to be sufficient for this study.

### Ethics

Ethical approval to conduct the study was obtained from the Institutional Ethical Review Board at R. D. Gardi Medical College, Ujjain, MP, India. Written informed consent was obtained from all respondents. Consent was administered in the local language by trained women data collectors. In case of illiterate participants, the consent form was read out and explained to them and on consenting they placed a thumb impression on the form instead of their signature. During analysis, data from participants and health facilities was anonymized, so that no individual or facility could be identified.

## Results

### Maternal characteristics

Mothers were young (mean age 23.5 years, SD 3.76), 41.5% had no education and 53.3% reported possessing a below-poverty- line card (BPL card) (Table 2). Most women had three or more antenatal care visits (75.7%), and about half were accompanied by ASHA during childbirth (Table 2). Seventy one percent lived ten km or less from their nearest OC facility. One in ten women lived near facility that provided cesarean section with or without blood transfusion services (Table 2).

**Table 2:** Women´s characteristics stratified by bypassing or not their nearest public health facility. n = 720.

Characteristics	No. (%)	Non bypasser	Bypasser	P-value*
	n = 720	n = 440	n = 280	Chi Square or T-test
		No. (%)	No. (%)	
**Maternal characteristics**				
Age in years (mean/SD)	23.5 (3.76)	23.6 (3.83)	23.4 (3.66)	0.48
Education. No / primary	299 (41.5)	197 (44.7)	102 (36.4)	0.02
Poverty (BPL) card. Yes	384 (53.3)	230 (52.3)	154(55.0)	0.47
Caste				0.89
ST	114 (15.8)	69 (15.7)	45 (16.1)	
SC	179 (24.9)	112 (25.5)	67 (23.9)	
OBC/General	427 (59.4)	259 (58.8)	168 (60.0)	
Primigravida	255 (35.4)	137 (31.2)	118 (42.2)	<0.001
**Health service utilization**				
At least three ANC visits	542 (75.7)	319 (72.5)	223 (79.6)	0.03
Accompanied by ASHA. Yes	345 (47.9)	215 (48.8)	130 (46.4)	0.52
Janani Express used. Yes	315 (43.7)	201 (45.6)	114 (40.7)	0.19
Hired transport used. Yes	255 (35.4)	132 (30.0)	123 (44.0)	<0.001
Complications during childbirth. Yes	101 (14.0)	42 (9.55)	59 (21.0)	<0.001
Distance from nearest facility (Km)				<0.001
< 5Km	247 (34.3)	199 (45.2)	48 (17.1)	
5 to 10 Km	263 (36.5)	141 (32.1)	122 (43.7)	
≥ 10 Km	210 (29.3)	100 (22.7)	110 (39.2)	
**Characteristics of nearest facility**				
Primary level Facility. Yes	329 (45.6)	144 (32.7)	185 (66.0)	<0.001
Secondary level facility. Yes	375 (52.0)	280 (63.6)	95 (34.00)	<0.001
Tertiary level facility. Yes	16 (2.2)	16 (3.6)	0 (0.0)	<0.001
Facility with cesarean section services. Yes	80 (11.1)	72 (16.4)	8 (2.9)	<0.001
Facility with blood transfusion services. Yes	52 (7.2)	50 (11.4)	2 (0.8)	<0.001
Number of basic signal function. Mean (SD)	3.27 (1.25)	3.55 (1.26)	2.8 (1.08)	<0.001
Free emergency referral transport. Yes	595 (82.6)	409 (93.0)	186 (66.5)	<0.001
At least one doctor. Yes	665 (92.3)	420 (95.5)	245 (87.5)	<0.001

*Difference between bypassers and non bypassers.

During the study period, most (94.8%) women delivered at a public health care facility, with 57% of all birth occurring in secondary health care facilities (Table 3) that had 31% of all available beds. In contrast, private health care facilities with 31% of all beds performed only 5.2% of all deliveries recorded (Table 3).

**Table 3:** Characteristics of the actual facilities where women gave birth in three districts of in Madhya Pradesh, India.

	Facility characteristics				
Type of the facility		Available beds	At least one doctor available (yes)	Free emergency transport services (yes)	Women giving birth
	n (%)	n (%)	n (%)	n (%)	n (%)
Tertiary	3 (3.1)	238 (22.3)	3 (100.0)	3 (100.0)	110 (15.3)
Secondary	23 (23.9)	331 (31.0)	22 (95.6)	19 (82.6)	415 (57.6)
Primary	48 (50.1)	168 (15.7)	38 (79.2)	26 (54.2)	158 (21.9)
Private	22 (22.9)	331 (31.0)	18 (81.8)	0 (0.0)	37 (5.2)

### Bypassing magnitude

As per our definition 38.9% (280) of the women whose nearest facility was public (n = 720) bypassed it. Mothers whose nearest OC facility was public travelled a median distance of 9.9 km (IQR 4.4–17.0 km) to reach their desired facility for childbirth. Bypassers travelled longer distances (median 18.4 km, IQR 14.4–24.4) than non bypassers (median 5.5 km IQR 2.7–6.9 km) (p < 0.001) (n = 720).

### What kinds of facilities were bypassed?

**Fig 1:**
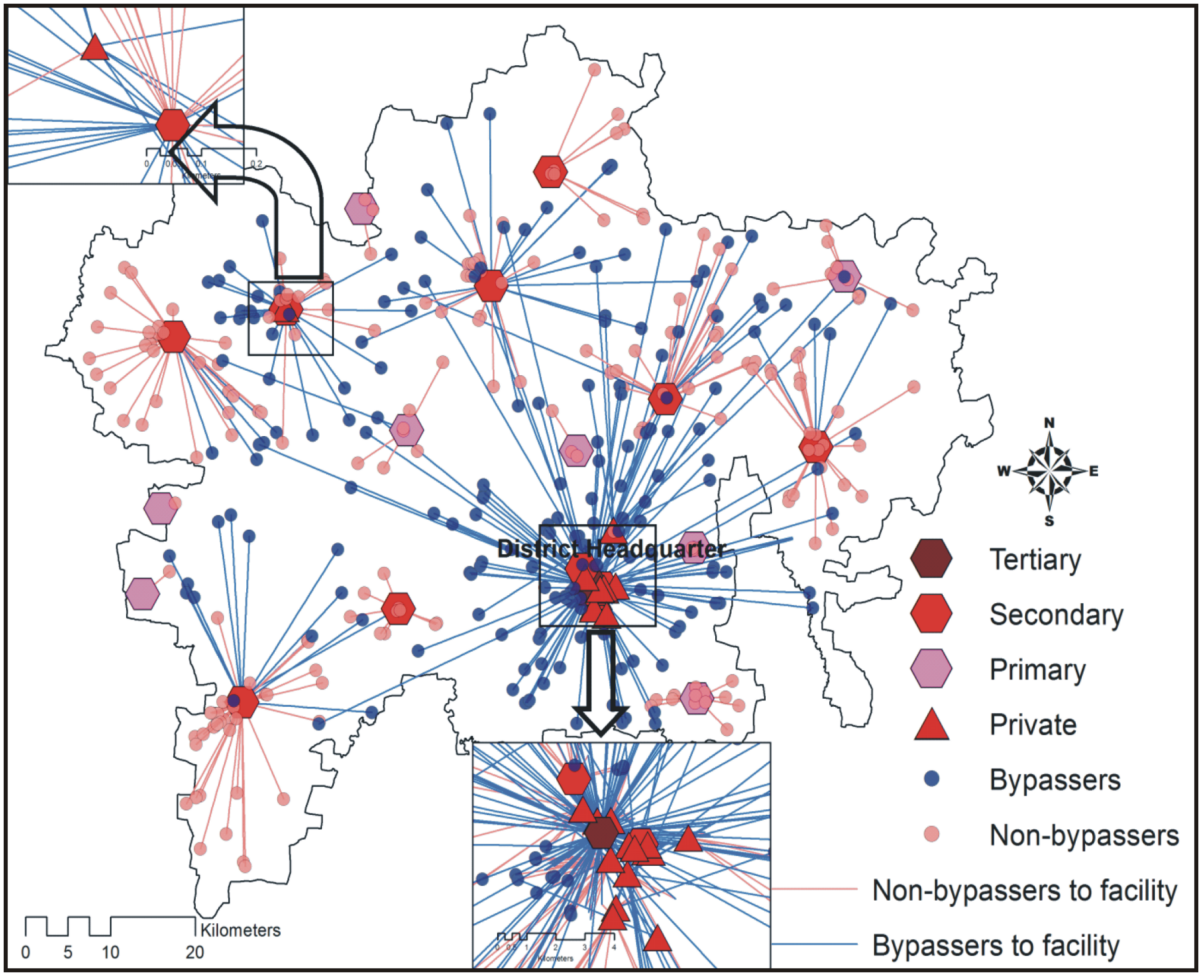
Mother´s bypassing patterns by obstetric care facility type in district 1.

**Fig 2:**
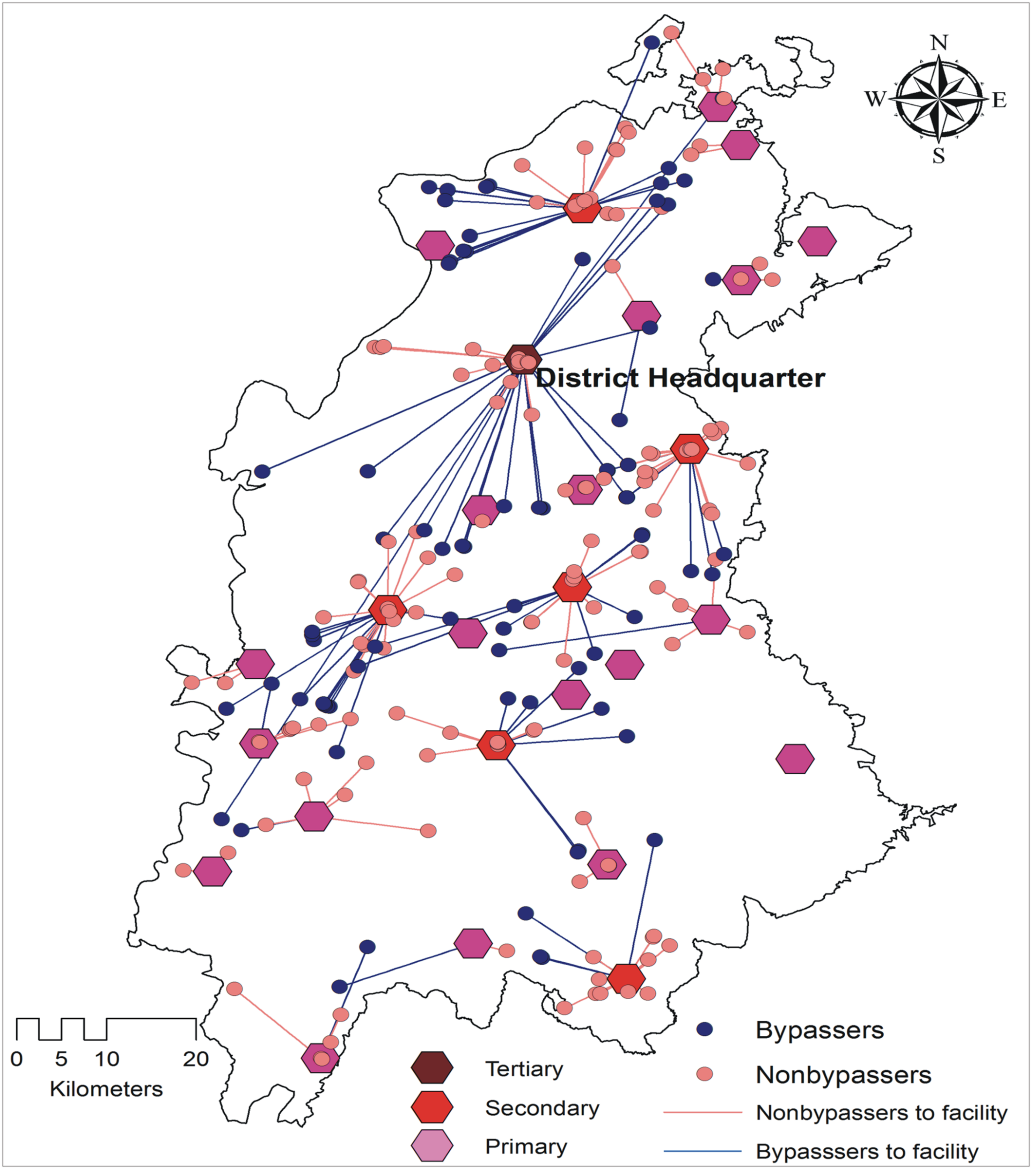
Mother´s bypassing patterns by obstetric care facility type in district 2.

**Fig 3:**
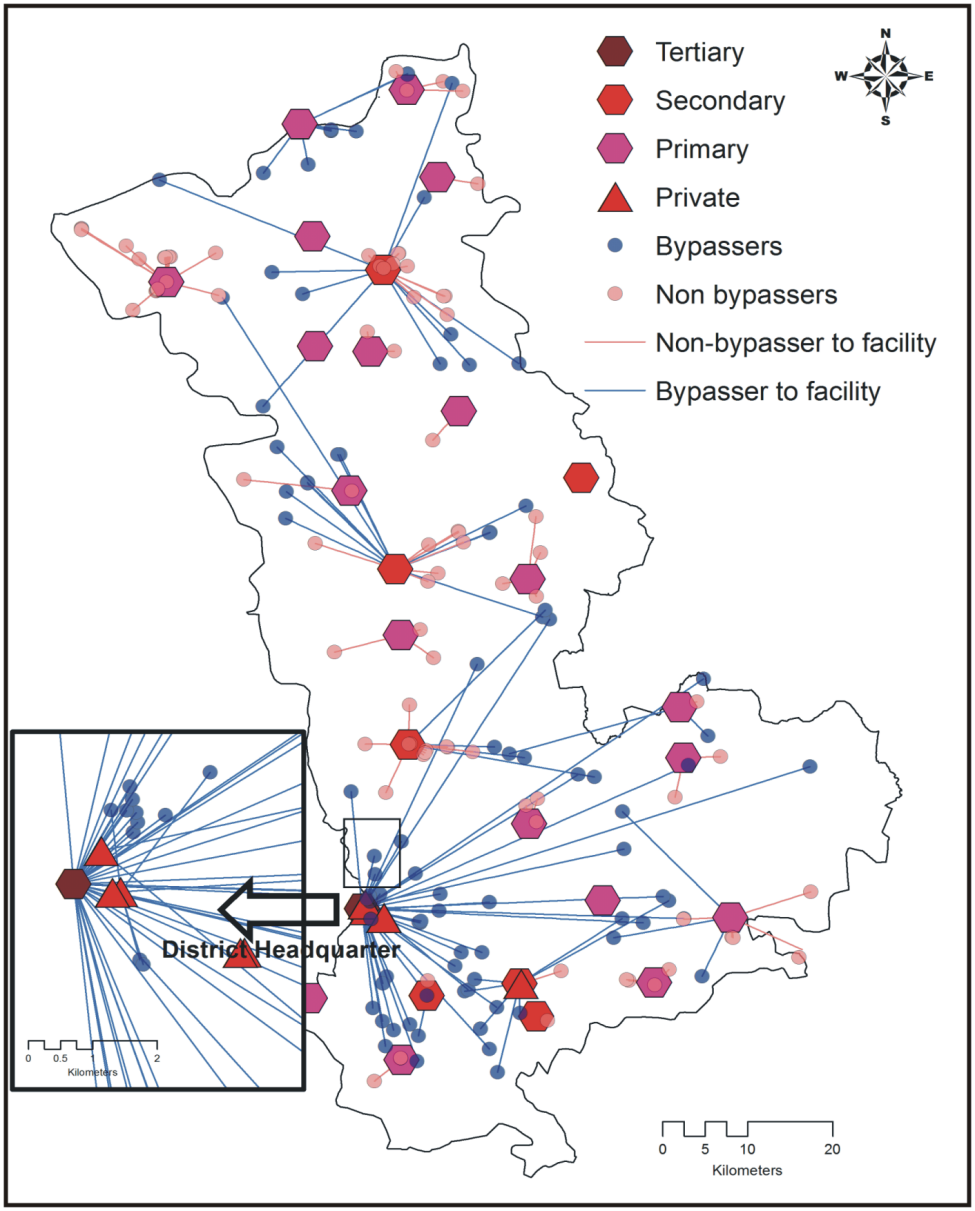
Mother´s bypassing patterns by obstetric care facility type in district 3.

Secondary and primary public health care facilities were the nearest OC facilities for most of the mothers (375 and 329 respectively, Table 4). Bypassing varied according to facility type. Public primary OC facilities were bypassed the most (56.2%, 185/329) followed by public secondary OC facilities. No tertiary public OC facility was bypassed (Table 4). Most women bypassing public facilities did so looking for care at a higher level public facility (Table 4). The patterns of bypassing are illustrated in the Figs 1, 2 and 3.

**Table 4:** Percentage of women who bypassed their nearest public OC facility by facility type, n = 720.

Type of facility	Number of nearest mother	Women who Bypassed facility	Facility of current delivery	No (%)
		No (%)	No (%)	
Tertiary (DH)	16	0 (0.0)	Tertiary	0 (0.0)
			Secondary	0 (0.0)
			Primary	0 (0.0)
			Private	0 (0.0)
Secondary (SDH/CHC)	375	95 (25.3)	Tertiary	47 (49.4)
			Secondary	29 (30.5)
			Primary	2 (2.1)
			Private	17 (17.8)
Primary (PHC /SC)	329	185 (56.2)	Tertiary	47 (25.4)
			Secondary	106 (57.3)
			Primary	19 (10.2)
			Private	13 (7.0)

### Differences between bypassers and non bypassers

Table 2 shows the proportion of bypassers and non bypassers among women whose nearest OC facility was public (n = 720). The proportion of bypassers was significantly higher among educated, primigravidas, those with more than three ANC visits, those who arrived by used a hired transport, resided more than 10km from their nearest facility, those who had any complication during childbirth, and among mothers whose nearest facility was a primary level facility (p<0.05). On the other hand, a higher proportion of non bypassers had a secondary level facility or tertiary level facility as their nearest facility, their nearest facility had free emergency referral transportation, or had at least one doctor (Table 3) (p<0.05). The proportion of mothers whose nearest facility had C-section or blood transfusion services was higher among non bypassers than among bypassers (p<0.05). Finally, the mean number of basic signal functions at the nearest facility was higher among non bypassers than bypassers (p<0.05, Table 2).

### A multilevel approach to the bypassing phenomenon

The multilevel analysis is shown in three models. Model one, the probability of bypassing the nearest facility is only a function of the facility itself, which is accounted for with a facility level intercept. The VPC of model one shows that 47% of the variance can be explained by the differences between OC facilities (Table 5). Model two, includes the women´s characteristics. This shows that having higher education, being a primigravida, having used hired transport to reach facility, having any complication during childbirth, and a long distance to the nearest facility were factors that increased the odds of women bypassing their nearest facility (p<0.05, Table 5).

**Table 5:** Multilevel logistic regression of the association between bypassing a facility, individual-level, and facility-level characteristics among women delivering in MP. Measures of association and clustering are shown. n = 720.

Measures of association (AOR, 95% CI)	1. Empty model	2. Model with individual-level variables		3. individual-level and facility-level variables	
***Individual-level variables***					
Education secondary/above (yes)	---	1.61	1.02–2.54	1.53	0.98–2.41
Primigravida (yes)	---	1.66	1.03–2.67	1.62	1.01–2.59
A least 3 antenatal care visits (yes)	---	1.61	0.92–2.81	1.91	1.10–3.33
Hired transport used (yes)	---	2.50	1.57–3.97	2.66	1.65–4.28
Complications during childbirth (yes)	---	4.99	2.60–9.60	5.14	2.69–9.82
Distance from nearest facility					
< 5km	---	1.00	1.00	1.00	1.00
5-10km	---	8.27	4.51–15.15	7.66	4.19–13.97
>10km	---	14.32	7.44–27.57	13.69	7.15–26.20
***Nearest Facility-level variables***					
Number of basic signal functions (unit)				0.59	0.37–0.93
C-sections last three months (yes)	---	---	----	1.14	0.04–27.32
Blood transfusion last three months (yes)				0.07	0.00–4.37
Free transportation (yes)	---	---	----	0.11	0.03–0.31
At least one doctor(yes)	---	----	----	0.72	0.16–3.19
Variance	3.01	4.60		2.00	
MOR*	5.25	7.74		3.58	
VPC‡	0.47	0.58		0.37	

*MOR = median odds ratio.

^‡^VPC = variance partition coefficient.

All models adjusted for all variables in the table.

Model three shows that after adjusting for all individual and facility variables included in the model, the individual factors associated with bypassing the nearest facility in model two remained significant (p<0.05, Table 5). At the facility level, the odds of bypassing the nearest facility decreased by 41% with an increase of one unit of the basic EmOC signal function scale (AOR = 0.59, 95% CI 0.37–0.93). Also, the odds of bypassing the nearest facility decreased by 89% if the facility had free transportation (AOR = 0.11, 95% CI 0.03–0.31). In model 3, the MOR shows that in the median case, the residual heterogenicity between facilities increased by 3.58 the odds of bypassing the nearest facility when randomly picking out two women with different facilities. The residual heterogeneity between facilities (MOR = 3.58) was more important than most of the mothers´ characteristics to explain the variation in the odds of bypassing a public facility for childbirth. Model 3 VPC shows that 37% of the variation in bypassing the nearest facility can be attributed to difference between facilities (p<0.05, Table 5).

## Discussion

This study showed that bypassing health facilities for childbirth in three districts of Madhya Pradesh, India is a common event. Our research is among the first papers from India to study the phenomenon of bypassing facilities for childbirth and to report on the importance of public facility functionality for the bypassing phenomenon. The findings also have relevance for the JSY program as discussed below.

### Individual factors associated with bypassing public health facilities under the JSY program

The overall bypassing figures in our study are similar as those reported by Kruk et al. in Tanzania (44%) [5], but lower than those found in Nepal (70%) [6], although different definitions of bypassing have been used by each of these studies. Besides the different bypassing definitions used, the variation between our figures and those estimated in Nepal could also be explained by several factors including differences in the relative availability, type, and geographic distribution of OC facilities.

Our data shows that ANC utilization was positively associated with bypassing. We argue that maternal contact with ANC services might have increased their awareness about complications during their current pregnancy that needed care not available at their nearest facility. The quality of the ANC provided at their nearest facility might have also influenced maternal decision to bypass it or not. For example, a study from Nepal found that frequent antenatal care visits were a protective factor against bypassing [6]. However, our study did not assessed whether mothers received ANC at their nearest facility or elsewhere, thus further studies are needed to support this hypothesis in this setting.

Our finding showing that peripartum complications were associated with a higher odds of bypassing facility are in line with the results of studies conducted in other low-income settings reporting positive associations between illness severity [1] or health complications [6] and bypassing. A number of complications in the peri-partum period might begin with the onset of labor before the woman leaves home. This could influence bypassing the nearest facility to seek better care.

Being a primigravida was another individual factor increasing the odds of bypassing, and this is line with findings from studies conducted in Nepal [6] and Tanzania [5]. It has been argued that women pregnant for the first time might be more anxious about the delivery than women who already had had at least one pregnancy, and this might influence their choice of health provider [6].

Our finding that using a hired transport to reach a health facility increased the odds of bypassing the nearest facility has not been reported before. In spite of the free emergency transportation JE service [22] to support the JSY program, another study has found that up to 38% of the pregnant women in our study setting used a hired transport to reach a facility for delivery [29]. Women with a better socioeconomic status can afford to hire transport which is relatively expensive; and can then direct the hired transport to take them to a health facility of their choice.

As found in other studies [5,30,31] distance was an important factor influencing women´s choice to bypass their nearest facility for delivery. Women who lived further away from the nearest peripheral facility had a higher odds of bypassing that nearest facility, as they had to travel far to reach that nearest facility, and so going to a higher facility further away needed little extra effort.

### Facility factors associated with bypassing public health facilities

Functionality indicators, such as the performance of EmOC signal functions, have been highlighted as key elements reflecting the ability of maternal health services to tackle maternal morbidity and mortality [23,32]; and thus can be used to measure the quality of maternal health services provided by health facilities. Researchers studying the bypassing phenomenon had identified that patient’s subjective perceptions of quality of care [5,6,31] and the facility’s general characteristics (i.e number of doctors, availability of drugs and equipment, availability of basic infrastructure) [1,2] as key factors associated with bypassing a health facility. Yet, studies assessing the relationship between OC signal functions and bypassing OC facilities for childbirth are rare [33], with few reported in India. Our multilevel study shows how a direct measure of functionality, the number BEmOC signal functions performed in a facility, decreased the odds of bypassing a facility even after adjusting for other facility and individual factors. These findings are especially relevant since 37% all the variation in this study was explained by the facility characteristics, and the variation between facilities (MOR = 3.85) was more important than most of the individual’s characteristics for explaining the bypassing phenomenon in this setting. The relevance of strengthening the availability of basic emergency obstetric care is vital if the phenomenon of bypassing for childbirth in MP is to be reduced. This is important if women are to receive adequate care under the JSY program, which has so successfully drawn mothers into public health facilities for childbirth.

### Findings´ relevance for the JSY program

These findings have relevance for the JSY program as they indicate a pattern of bypassing resulting in underuse of public lower level facilities. This might have negative impacts on care at all facilities levels. At the lower levels, fewer cases will mean a loss of skills, and a diminishing ability to provide BEmOC signal functions. On the other hand, bypassing primary and secondary obstetric care centers might overburden tertiary level facilities, stressing the limited staff and resources which can have a negative effect of the quality of care provided [8]. Thus, it is an issue of concern that in our study nine out of ten women delivering at the three public tertiary level facilities bypassed another facility, with 64% of those bypassing primary or secondary public facilities.

Pregnant women´s choice to bypass lower level facilities might be related to their perception of the quality of care received [2,5,6,31]. These perceptions are supported by our work and by other studies in this setting which reported that nurses (the main cadre of staff providing obstetric care at primary and secondary levels) have low competence in providing critical obstetric care [34,35]. Our findings indicate the need for strengthening functionality at the lower level facilities and possibly increasing staff and capacity at the higher levels if the increase in institutional delivery is to be spread more equally across all levels of the public health system.

### Strengths and limitations

Multilevel studies assessing the bypassing phenomenon are rare. We only found one multilevel study conducted in Tanzania showing that 16% of the variation was explained by different perceptions of quality aggregated at the village level [36]. In contrast, our study found that 35% of the overall variance was explained by specific measures of quality (functionality) at the facility level witch might be more useful to stakeholders to implement relevant changes in the health system.

This study has some limitations that are important to acknowledge. Our study did not measure individual perceptions of quality which has been shown to be associated with the bypassing phenomenon [2,31,36], yet we used a more objective measure of quality (EmOC signal functions) that could be related to the individual perceptions of it. Another important limitation is that our study did not assessed whether facilities with high obstetric functionality were overcrowded. The cross-sectional design of this study did not allowed us to estimate whether the bypassing phenomenon has changed since the implementation of JSY program.

## Conclusions and recommendations

Bypassing health facilities for childbirth can be costly both for women and for the health system. These inefficiencies are likely to be multiplied in the context of the JSY program which has been extremely successful at drawing millions of women into public facilities to give birth. These findings have relevance for the JSY program as they indicate underuse of lower level facilities. Our findings indicate that functionality of a facility is a key determinant of it being bypassed or utilized. Focusing on strengthening this functionality will result in more efficient program and health system.

## Abbreviations

ANC: Antenatal care
ASHA: Accredited social health activist
BEmOC: Basic emergency obstetric
BPL: Below-poverty- line certification card
CEmOC: Comprehensive emergency obstetric
CHC: Community health centers
DH: District hospitals
IMR: Infant mortality rate
JE: Janani Express
JSY: Janani Suraksha Yojana program
MMR: Maternal mortality ratio
MOR: Median Odds Ratio
MP: Madhya Pradesh
OC: Obstetric care
PHC: Primary health centers
REDCap: Research Electronic Data Capture
SC: Sub centers
SDH: Sub-district hospitals
VPC: Variance partition coefficient
